# Validation of the Recent Life Changes Questionnaire (RLCQ) for stress measurement among adults residing in urban communities in Pakistan

**DOI:** 10.1186/s40359-019-0341-9

**Published:** 2019-10-21

**Authors:** Azmina Artani, Ayeesha K. Kamal, Syed Iqbal Azam, Moiz Artani, Shireen Shehzad Bhamani, Mehreen Saif, Fariha Afzal Khan, Nazir Alam

**Affiliations:** 1 Stroke Service, Section of Neurology, Department of Medicine, Aga Khan University, Karachi, Pakistan; 2 Stroke Fellowship Program, International Cerebrovascular Translational Clinical Research Training Program, Fogarty International Center and the National Institute of Neurologic Disorders and Stroke, Aga Khan University, Stadium Road, Karachi, 74800 Pakistan; 3 Biostatistics and Epidemiology, Department of Community Health Sciences, Aga Khan University, Karachi, Pakistan; 4 MBBS Program, Jinnah Medical and Dental College, Karachi, Pakistan; 5 Aga Khan University School of Nursing & Midwifery, Karachi, Pakistan; 6 Bahria University, Karachi, Pakistan; 7 University of Karachi, Karachi, Pakistan

**Keywords:** Stress, Mental disorders, Validation, RLCQ, Developing countries

## Abstract

**Background:**

Recent Life Changes Questionnaire (RLCQ) developed by Richard Rahe has enabled quantification of stress by analyzing life events. The overall aim of the study was to create a reliable version of the Rahe’s RLCQ for measuring stress in individuals living in developing countries and assess its validity. This paper discusses criterion validation of the adapted RLCQ in urban communities in Pakistan.

**Methods:**

This is a criterion validation study. Four urban communities of Karachi, Pakistan were selected for the study in which households were randomly chosen. Two data collectors were assigned to administer the adapted RLCQ to eligible participants after obtaining written informed consent. Following this interaction, two psychologists interviewed the same participants with a diagnostic gold standard of Mini International Neuropsychiatric Interview (MINI) which is utilized in usual practice within Pakistan to confirm the presence of stress related mental disorders such as Depression, Anxiety, Dysthymia, Suicide, Phobia, OCD, Panic Disorder, PTSD, Drug abuse and dependence, Alcohol abuse and dependence, Eating Disorders and Antisocial Personality Disorder to validate the accuracy of the adapted RLCQ. We generated the ROC curves for the adapted RLCQ with suggested cut-offs, and analyzed the sensitivity and specificity of the adapted RLCQ.

**Results:**

The area under the receiver operating characteristic curve (ROC) of common mental disorders such as depression and anxiety was 0.64, where sensitivity was 66%, specificity was 56% and the corresponding cut off from the adapted RLCQ was 750. Individuals scoring ≥750 were classified as high stress and vice versa. In contrast, the area under the ROC curve for serious mental disorder and adverse outcomes such as suicide, bipolar and dysthymia was 0.75, where sensitivity was 72% and specificity was 60% at the cut off of 800 on the adapted RLCQ. Individuals scoring ≥800 were classified as high stress and vice versa. The rate of agreement between the two psychologists was 94.32% (Kappa = 0.84).

**Conclusion:**

The adapted and validated RLCQ characterizes common mental disorders such as depression and anxiety with moderate accuracy and severe mental disorders such as suicide, bipolar and dysthymia with high accuracy.

**Trial registration:**

Clinicaltrials.gov NCT02356263. Registered January 28, 2015. (Observational Study Only).

## Background

Stress influences the capacity of a human to adapt to perceived or real life changes and has a major impact on physical and mental well-being of an individual [1]. Stress can be defined as a perceived loss of an individual’s ability to adapt with evolving changes in life [2]. Stress is commonly mediated by repeated Stressful Life Events in the areas of health, work, and environment, personal and social life of an individual [3–5]. Measuring stress accurately in community settings is a challenge, however, chronic stress from which the individual subsequently fails to cope, becomes apparent in the form of common mental disorders [6]. Common mental disorders generally refers to depressive disorders (major depression, dysthymia, and mood disorders such as bipolar affective disorder and mania), anxiety disorders (generalized anxiety disorder, phobias, post-traumatic stress disorder and obsessive-compulsive disorder), and suicide [6, 7]. Globally, common mental disorders are the leading cause of years lived with disability where more than 80% of disease burden is borne by low-middle income countries [7]. Findings from a meta-analysis of available data around the world from 1980 to 2013 suggested that at least one in five adults reports experiencing a common mental disorder within past 12 months while approximately 30% report suffering from it across their lifetime [8]. In Pakistan, studies conducted in different urban and rural areas have reported around 10–40% of affected individuals with depression indicating a high burden of underlying stress among people [9, 10].

Rahe and colleagues made an effort to quantify stress and developed a stress measurement scale namely Recent Life Changes Questionnaire (RLCQ) in 1996 [11, 12]. The RLCQ includes a list of probable daily life stressors that one goes through in five major life domains i.e. home, health, work, financial and social life of a person [11]. There are a total of 74 life events listed in the original RLCQ, that assigns a numerical value to each life event with cut-offs indicating high recent life stress. The RLCQ was developed and validated in a developed world setting and does not reflect the contextually relevant stressors of populations that reside in the developing world [13–18]. Attached is a review of previous validation and adaptation studies conducted on stressful life events in a global context (Additional file 1).

The overall aim of our study was to create a reliable adaptation of the Rahe’s RLCQ to make it appropriate for measuring stress in persons living in a developing country like Pakistan and assess its criterion validity. The first step was to create an adapted version of the RLCQ via iterative qualitative interviews and these results have been published [19]. After adaptation, we validated the adapted RLCQ comparing it with a diagnostic standard; the MINI International Neuropsychiatric Interviews (MINI).

MINI is a structured, diagnostic tool that recognizes individuals with mental illnesses and captures a broad spectrum of mental disorders including depressive disorders, anxiety disorders and suicidality [20]. It is deemed as an appropriate and a useful tool in detecting common mental illnesses in community based, primary health care setting and research [21, 22]. Globally, MINI has been used as a diagnostic standard in validation studies assessing performance of other measurement scales (self-reported or interviewer administered) identifying stressful life events and common mental illnesses across different populations [15, 23–25]. In addition, it is widely used in Pakistan by psychologists and psychiatrists for clinical decision-making and diagnosis making it acceptable to be used as a diagnostic standard to compare the performance of adapted RLCQ in capturing mental illnesses [26].

This paper discusses criterion validation of the adapted RLCQ in urban communities in Pakistan and reports its measures of validity i.e. sensitivity and specificity of the adapted RLCQ.

## Methods

### Study design

This is a criterion validation study where our aim was to validate the adapted RLCQ by comparing it with a diagnostic parameter (gold standard) practiced in Pakistan that measures the construct related to stress. Hence, the concept of recent life changing events was validated by comparing it with a diagnostic tool of mental illness to assess the extent of accuracy in measurement by exploring the agreement between them [26]. The data was collected in a cross-sectional way by administering the adapted RLCQ and the gold standard simultaneously at one point in time. Furthermore, we performed exploratory analysis on the effect of resilience on stress level and mental illness in the community.

### Gold standard

The Mini International Neuropsychiatric Interviews (MINI) is a standardized, structured diagnostic interview based on the DSM-IV and ICD-10 criteria [20]. It is easy to administer, takes moderate time (approximately 45 min) and is used in psychiatric practice with in Pakistan for diagnosis of a broad spectrum of common mental illnesses [27]. MINI’s scope covers Depression, Dysthymia, Suicide, Phobia (Social and Agoraphobia), Obsessive Compulsive Disorder (OCD), Panic Disorder, Post-Traumatic Stress Disorder (PTSD), Drug abuse and dependence, Alcohol abuse and dependence, Eating Disorders (Anorexia and Bulimia Nervosa), Generalized Anxiety Disorder (GAD) and Antisocial Personality Disorder [20].

### Study sites and population

Four urban communities in Karachi namely Kharadar, Dhorajee, Gulshan and Garden were selected. The sites selected for this phase were the same as that of the adaptation phase of the RLCQ. Adults aged 18 years or more living in these communities in Karachi who fulfilled the eligibility criteria were invited to become part of the study upon obtaining written informed consent. We excluded individuals who did not understand Urdu, had cognitive or hearing difficulties and had known psychiatric illnesses which would impair their understanding of the questions posed by the interviewers.

### Sample size and sampling technique

A sample of 300 participants was required to achieve at least a power of 80% at a level of significance of 5% to detect a two sided difference of 0.1 from area under the curve of Receiver Operating Characteristic curve of 0.80. The prevalence of mental illnesses in Karachi as reported in literature was taken to be 20% which was done in an effort to capture cases as well as non-cases in our sample [10]. Software NCSS-PASS version 11.0 was used for this sample size calculation.

For sampling, we utilized list of households in these areas and excluded the ones selected in the adaptation phase. Households were chosen randomly identifying one member from each household.

### Administration of adapted RLCQ and MINI

**Fig. 1:**
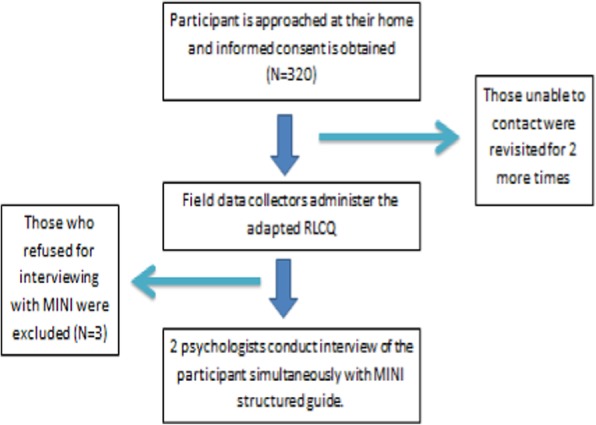
Flow Diagram of Validation Phase

There were two data collection teams. Each team comprised of two trained field officers and two psychologists. Trained Field officers were responsible to approach participants, determine eligibility and seek informed consent. Then, they administered the adapted RLCQ to the study participants after which psychologists interviewed them based on MINI. The psychologists conducted MINI interviews together but they were not allowed to see or discuss their evaluations. Hence, their decisions were independent of each other. In case of discrepancy in diagnosis of the psychologist, opinion was sought from another expert (Fig. 1).

For quality control, a supervisor accompanied study teams into the community. Spot-checks were done to verify quality and accuracy of the information. Field officers were instructed to minimize their interaction with the psychologists so as to avoid data contamination.

### Human subjects approvals and registration

The study received ethical approval from Ethical Review Committee, Aga Khan University on 14th October 2014 with study registration ID as 3235-CHS-ERC-14. The study is also registered as an observational study at Clinicaltrials.gov with the study ID NCT02356263. All study team members received additional training and certification on research ethics prior to starting this community based study. Since we interviewed participants at their home, special measures were taken to maintain confidentiality of the participants from other members of the household. Counseling sessions were also conducted by these psychologists upon identifying individuals having mental illnesses. Also, referrals of psychologists were given for further consultation. Participants were also provided with study help line numbers if they wanted to contact study team or psychologists at any time during the entire study period.

### Statistical analysis plan

Statistical analysis was performed using software STATA version 12. Means and proportions were calculated for baseline characteristics of the study population. A composite score was generated by adding scores of every event on adapted RLCQ. The receiver operating characteristic curve (ROC) was formed and its area under the curve (AUC) was determined for each of the mental illnesses that MINI caters, to obtain the composite score on the adapted RLCQ. Categories of low and high stress were subsequently made based on the chosen cut-off with the help of ROC curves and sensitivity and specificity of the adapted RLCQ were evaluated. Also, while we were relying on the judgment of two psychologists, we applied Kappa Statistic to determine the extent of agreement between them. A Kappa statistic of 0.60–0.80 was considered as substantial agreement while 0.81 or higher was considered near perfect agreement.

## Results

The mean age of our study participants (*n* = 317) was 46.65 ± 9.3 years. Of the total, 87% of the sample was married and 76% of the population was of females. 50% of the sample had at least received secondary level of education (Table 1).

**Table 1:** Baseline characteristics of participants in validation phase

Baseline Characteristics of Participants	N (%)
Age^a^ (years)	46.65 (9.32)
Gender	
Male	76 (24%)
Female	241 (76%)
Marital status	
Single	14 (4%)
Married	277 (87%)
Divorced/separated/widowed	25 (8%)
Education status	
No formal education	89 (28%)
Madrasa	5 (2%)
Primary level	44 (20%)
Secondary level	112 (50%)
Intermediate and above	68 (31%)
Family’s monthly income (PKR)^b^	Median 19,000 (IQR 12,000- 25,000)

^a^Mean (Standard Deviation)

^b^Median (Interquartile Range)

From our sample, the overall estimate of the prevalence of mental disorders in Karachi is 26.5% with depression (9.2%), Drug abuse and dependence (5.7%), Suicide (5.4%), Dysthymia (4.4%) and GAD and PTSD (2.8%) as the most common ones (Table 2).

**Table 2:** Prevalence of mental disorder in Karachi

Mental Disorders	n (%)
Depression	29 (9.2%)
Suicide	17 (5.4%)
Dysthymia	14 (4.4%)
Generalized Anxiety disorder (GAD)	9 (2.8%)
Post-Traumatic Stress Disorder (PTSD)	9 (2.8%)
Panic Disorder	6 (1.9%)
Obsessive Compulsive Disorder (OCD)	5 (1.6%)
Antisocial Personality Disorder	0
Abuse and Dependence	
Drugs	18 (5.7%)
Alcohol	0
Phobia	
Agoraphobia	2 (0.6%)
Social	1 (0.3%)
Bipolar	
Hypomania	3 (1.0%)
Mania	2 (0.6%)
Eating Disorder	
Bulimia Nervosa	1 (0.3%)
Anorexia Nervosa	0
All Mental Disorders	84 (26.5%)

For a ROC curve, AUC provides a snapshot of the ability of a screening tool in capturing true positive rate (y-axis) in contrast to false positive rate (x-axis: 1-specificity). Upon careful examination of these curves, we were able to classify them into three levels i.e. disorders having an AUC of ≤0.5, disorders having an AUC of > 0.5 and < 0.7 and disorders having an AUC of ≥0.7. Of all the mental disorders, we were not able to formulate ROC curves of Alcohol abuse and dependence, antisocial personality disorder and Anorexia nervosa because we did not encounter any of the participants with these disorders in our sample. We categorized related disorders under one umbrella such as eating disorders for Anorexia and bulimia nervosa. A similar approach was used for phobias, drug abuse and dependence and bipolar disorder (Table 3).

**Table 3:** Mental disorders and their respective area under the curve of ROCs

Mental Disorders (AUC)		
AUC ≥ 0.7 (Good)	0.7 > AUC > 0.5 (Moderate)	AUC ≤0.5 (Poor)
Bipolar (0.78)	PTSD (0.67)	OCD (0.5)
Dysthymia (0.76)	Eating Disorder (0.63)	Drug abuse and dependence (0.44)
Suicide (0.72)	Phobia (0.56)	Panic Disorder (0.42)
	Depression and Generalized Anxiety Disorder (GAD) (0.54)	

We identified 3 disorders which our adapted tool classified with good accuracy (Table 3). We formulated a category of “Common Mental Disorders (CMD)” where in addition to these three disorders we included Depression and Generalized Anxiety Disorder (GAD) due to their high prevalence and public health importance.

**Fig. 2:**
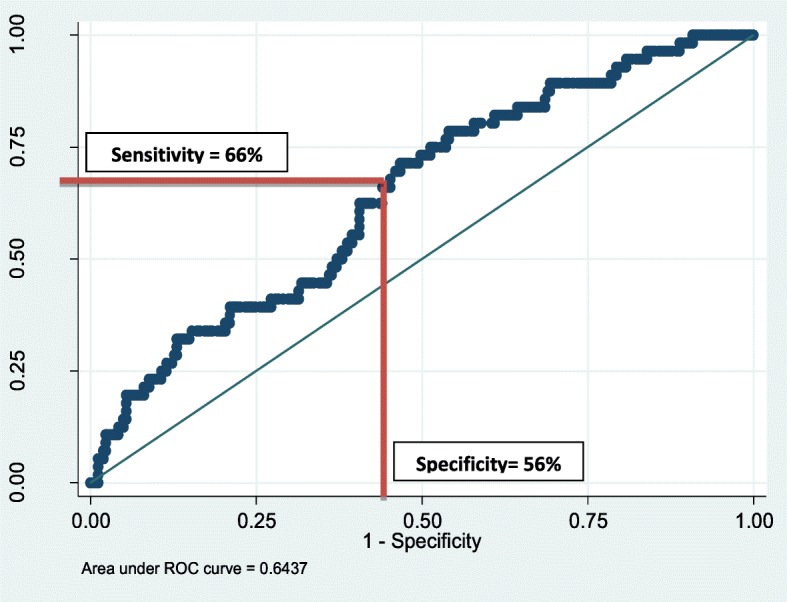
ROC curve of Common Mental Disorders (Bipolar, Dysthymia, Suicide, Depression and GAD)

The AUC of common mental disorders was 0.64, where sensitivity was 66% and specificity was 56% and the corresponding cut off from the RLCQ was 750. We chose for a cut-off that attains a maximum balance between sensitivity and specificity. In situation of discrepancy, a higher sensitivity was preferred to that of specificity as we desired to make adapted RLCQ as a community screening tool for stress [28]. At this level, the trade-off between sensitivity and specificity was the least. As we aim at making our adapted RLCQ as a screening tool for stress, a higher sensitivity was more meaningful to us than specificity [28]. Therefore, any individual having a composite score on the adapted RLCQ ≥750 will be classified as highly stressed; on the contrary, those having a composite score of < 750 on adapted RLCQ will be classified as low stress for development of CMD as identified above (Bipolar, Dysthymia, Suicide, Depression and GAD) (Fig. 2).

**Fig. 3:**
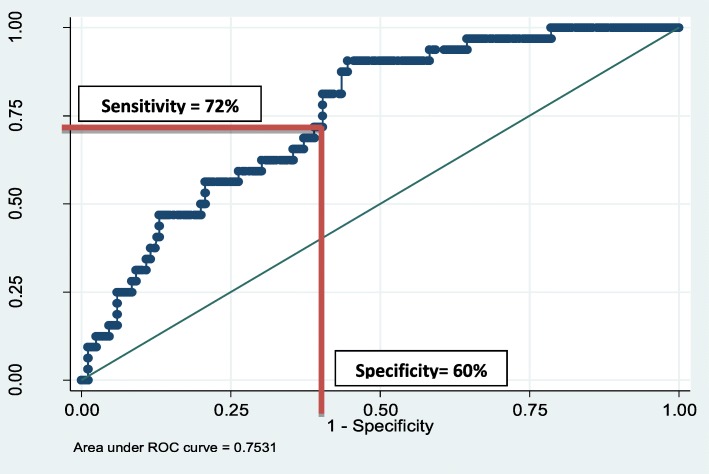
ROC curve of Serious Mental Disorder and Adverse Outcomes (Bipolar, Suicide and Dysthymia)

Similarly, based on this study results, we formulated a category of “Serious Mental Disorder and adverse outcomes” encompassing Bipolar, Suicide and Dysthymia. The AUC of Serious Mental Disorder and Adverse Outcomes was 0.75, where sensitivity was 72% and specificity was 60% at the cut off of 800 on the RLCQ (Fig. 3).

The agreement between the two psychologists that were making judgments about the presence of mental illnesses was 94.32%. Kappa statistic of the inter-rater agreement was 0.84 (standard error 0.05).

### Exploratory analysis of factors affecting ROC predictability

Based on our observations, we considered “Resilience” to be playing an important role in the relationship of stress and occurrence of mental illnesses. Resilience is the ability of an individual to cope from stressful situations occurring in life and may vary from individual to individual [29]. Conceptually, those individuals who had a composite score of ≥750 on the adapted RLCQ, due to having a high resilience, might not have had a mental illness. To explain the mechanism of this effect, we collected data on the resilience of all the individuals in our study using Urdu version of Wagnild’s Resilience Scale which was validated in Pakistan [30, 31]. We found that among those individuals who had low resilience, the odds of getting a common mental disorder is 3.4 times with high stress as compared to low stress (*p*-value = 0.01, 95% CI = 1.34–8.8). This association augments the fact that despite lower AUC of ROC of the adapted RLCQ, it is because of an intrinsic factor of the participants themselves (resilience) rather than the capacity of the tool itself to predict the absence and presence of mental disorders.

## Discussion

The results of this validation study enabled us to identify people experiencing high stress and having the potential of developing serious mental disorder and adverse outcomes like suicide, bipolar and dysthymia with high accuracy, and the potential development of common mental disorders like depression and anxiety with moderate accuracy in our urban multiethnic communities. It does not however allow us to predict the development of a mental disorder over time as this is not a longitudinal study and informs stress related to having a mental condition. We cannot use the scale for OCD, Panic Disorder, Eating Disorder, Drug and alcohol dependence, as these observations were limited to inform validation. We also have defined ROC based cut offs for high and low stress scores with reasonable sensitivity and specificity. Those scoring RLCQ at 750 or greater are 75% likely to be screened appropriately for common mental disorders and serious mental disorder and adverse outcomes like suicide, bipolar and dysthymia.

Pakistan being a resource strapped low middle-income country (LMIC) faces a huge challenge to provide health care to its population. Generally, resources for the provision of mental health care are very limited such that only 2–3 psychiatrists are available per million population [32, 33]. A possible solution is a community mental health approach. Trained community health workers have been effective as task shifters to provide health care at a population level [34, 35]. Studies done in the South Asian region promises better outcomes for community via efficient mental health training programs for CHWs [36, 37]. Our aim of adapting and validating RLCQ was to develop a screening tool for mental health which enable the capacity to measure stressors of the population accurately and identify those who may be experiencing higher level of stress. The adapted RLCQ is a simple tool and does not require highly qualified individuals to administer. In this study, adapted RLCQ was administered by trained field workers with a basic level of education and thus, it can be administered by the community health workers easily upon training who are equivalent to field workers in our study. In addition, it takes less time than psychological surveys. The maximum duration of administration of the adapted RLCQ had been 20 min. This approach may assure delivery of mental health facilities embedded in the primary health care model within Pakistan and may increase uptake of these services when provided by their own community involved workers.

Our adapted RLCQ mirrors stressful events in context of the study population as it is a community based study. The magnitude of each event was derived from household surveys with community input. We targeted study sites which represented urban individuals and can be generalizable to the urban population of Pakistan at large except for overseas Pakistanis as some of the environmental factors would have been modified depending on their country of residence. We kept adapted RLCQ more sensitive than specific so as to have minimum loss to capture of those who are stressed and are vulnerable to mental illness. However, an AUC of 0.64 makes it moderately accurate for certain mental illnesses. We explored the biological underpinnings of why an adapted and contextually relevant RLCQ would not predict the development of mental illness and we explored resilience as a modifier. Resilience exploration revealed how powerful it can be in modifying stress outcomes in terms of development of mental illness especially depression and GAD.

The study has certain limitations. There are stressful life events that will not be discussed in any research or public context due to stigma or taboos. These may include sexual violence, alcohol or drug abuse for example. There may be some element of recall bias as it is inherent in the cross-sectional design of study, however as these events are objectively occurring in the life of an individual, the chance of recall bias is minimal. For any country with socio-political instability, it is highly possible that new events would occur in a short time frame that would affect validation and retest reliability. Also, there may be individuals who may have experienced stressful life events but because of their high resilience may not necessarily have ended up with mental illness, thus we acknowledge this limitation. Additionally, our rationale to take presence of mental disorder as a criterion was to be able to state that a level of quantifiable stress has resulted in an adverse outcome and we tried to correlate where in the scale a mental disorder appears (at what level of experience of stressful life events, or score). These are the pragmatic rationales and considerations used in other studies as well, however other gold standards could also be used for validation depending on the context. Literature suggests that chronic stress may contribute to the development common mental disorders including depressive disorders, anxiety disorders, suicide and other mental illnesses. It is this aspect of the MINI that we have used. MINI covers a broad spectrum of mental illnesses including post-traumatic stress disorders. This is the rationale of choosing this as the gold standard in addition to the fact that it has been used in these settings, however other standards can also be utilized in this context which may be associated with more robust predictions. Future research may look into the role of resilience in modifying stress experience and its measurement in the population, future research directions may also cover better elaboration of stress experience.

## Conclusion

The outcome of this study provides the validated tool that can be used as a community mental health intervention with its inherent strengths and limitations. We recommend that future studies should explore test-retest studies for different users and to examine the effects of resilience on stress and resilience boosting strategies in marginalized and vulnerable urban populations.

## Supplementary information

**Additional file 1.** Validation Studies Conducted for SRRS, SLE and RLCQ.

## Data Availability

All data generated or analyzed during this study are included in this published article. Further material details are available from the corresponding author on reasonable request.

## Abbreviations

AUC: Area under the curve of ROC
CMD: Common mental disorder
DSM IV: Diagnostic and Statistical Manual of Mental Disorders, 4th Edition
GAD: Generalized anxiety disorder
ICD 10: International Statistical Classification of Diseases and Related Health Problems (ICD)
LMIC: Low-middle income countries
MINI: Mini International Neuropsychiatric Interviews
OCD: Obsessive compulsive disorder
PTSD: Post-traumatic stress disorder
RLCQ: Recent Life Changes Questionnaire
ROC: Receiver Operating Characteristic curve
SMD: Serious Mental Disorder and Adverse Outcomes
