# Supplementary material for: Validation of the Recent Life Changes Questionnaire (RLCQ) for stress measurement among adults residing in urban communities in Pakistan
Source: BMC Psychol. 2019 Oct 21;7:66. doi: 10.1186/s40359-019-0341-9 (PMC6805381; doi:10.1186/s40359-019-0341-9)
Supplement: Supplementary file 1 — Additional file 1. Validation Studies Conducted for SRRS, SLE and RLCQ. [file 40359_2019_341_MOESM1_ESM.pdf]

Appendix 1: Validation Studies Conducted for SRRS, SLE and RLCQ [1-7]

Authors [Scales] (Reference)	Population	Validated against/Outcomes	Year	Country	Sample Size
**Linden W. [SSRS] (1)**	College students (17-25 years)	Failed courses, had personal illness or injury or sought psychological help	1984	Canada	225
**Arias et al. [RLCQ Spanish Version] (2)**	Adults (20-50 years)	Adaptation, Translation into Spanish, Reproducibility	1999	Venezuela	60
**Ovuga E, Boardman J, Wasserman D. [RISLE-I] (4)**	Adults community participants and University students	Concurrent Validity using Beck Depression Inventory and Beck Scale for Suicide Ideation	2005	Uganda	1039
**Ovuga E, Boardman J, Wasserman D. [RISLE-II] (4)**	Adults community participants and University students	Criterion validity: Clinical interviews with Mini International Neuropsychiatric Interviews (MINI)	2005	Uganda	125 (Sub-sample of RISLE-I)
**Roohafzah et al. [SLE] (5)**	Adults	Validity: General Health Questionnaire (GHQ-12), Reliability	2011	Iran	3,9551
**Sali et al. [SLE-revised] (6)**	Adults (18-85 years)	Validity: GHQ-12 and artificial intelligence testing	2013	Iran	4,569

**Rios MR at al.** **[SLE] (7)**	Spanish speaking Peruvian adults	Criterion validity: Hopkins Symptom Checklist-25 (anxiety, depression and general distress)	2014	Peru	844

References:

1. Linden W. Development and initial validation of a life event scale for students. Canadian Journal of Counselling and Psychotherapy/Revue canadienne de counseling et de psychothérapie. 1984;18(3).
2. Arias I, Rodriguez E, Padilla J, Gonzalez N, Rodriguez MA. Translation to Spanish, reproducibility, and cross-cultural adaptation of the Miller-Rahe recent life change questionnaire in Venezuela. Arthritis & Rheumatism. 1999;12(4):287-93.
3. Ovuga E, Boardman J, Wasserman D. The response inventory for stressful life events (RISLE) I. refinement of the 100-item version. African health sciences. 2005;5(2):137-44.
4. Ovuga E, Boardman J, Wasserman D. The response inventory for stressful life events (RISLE) II: validation of the 36-item version. African health sciences. 2005;5(2):145-51.
5. Roohafza H, Ramezani M, Sadeghi M, Shahnam M, Zolfagari B, Sarafzadegan N. Development and validation of the stressful life event questionnaire. International journal of public health. 2011;56(4):441-8.
6. Sali R, Roohafza H, Sadeghi M, Andalib E, Shavandi H, Sarrafzadegan N. Validation of the revised stressful life event questionnaire using a hybrid model of genetic algorithm and artificial neural networks. Computational and mathematical methods in medicine. 2013;2013.
7. Morote Rios R, Hjemdal O, Martinez Uribe P, Corveleyn J. Life stress as a determinant of emotional well-being: development and validation of a Spanish-Language Checklist of Stressful Life Events. Health Psychology and Behavioral Medicine: an Open Access Journal. 2014;2(1):390-411.
